# Hypertrophic Cardiomyopathy Requiring Myectomy in a Young Patient With Systemic Lupus Erythematosus: A Case Report

**DOI:** 10.7759/cureus.61976

**Published:** 2024-06-08

**Authors:** Wael Abdelmottaleb, Donclair Brown, Mustafa Ozbay, David Rubinstein, Rosy Thachil

**Affiliations:** 1 Internal Medicine, New York Medical College/Metropolitan Hospital Center, New York, USA; 2 Internal Medicine, North Central Bronx/Jacobi Medical Center, New York, USA; 3 Cardiology, Elmhurst Hospital/Mount Sinai College of Medicine, New York, USA

**Keywords:** systemic lupus erythematosus (sle), hypertrophic cardiomyopathy, septal myectomy for hocm, sle and lupus nephritis, hypertensive emergency

## Abstract

Systemic lupus erythematosus (SLE) is an autoimmune disease that can affect multiple organ systems. Among these, the heart, including the pericardium, conduction system, myocardium, valves, and coronary arteries, can be affected. Hypertrophic cardiomyopathy (HCM) is a myocardial disease caused mainly by genetic mutation. The association between SLE and HCM is still unclear.

We are reporting a case of a 25-year-old female with SLE with end-stage renal disease (ESRD) due to lupus nephritis, who was found to have hypertrophic obstructive cardiomyopathy (HOCM) on the echocardiogram and required septal myectomy. She presented to the hospital with dyspnea and was admitted as a hypertensive emergency with pulmonary edema, which required intubation and admission to the cardiac intensive care unit (CICU). She underwent urgent hemodialysis and blood pressure medication adjustment and then improved and was discharged home.

Based on the literature review, 10 cases of SLE and HCM were reported, and the underlying mechanisms linking SLE and HCM remain unclear. Further studies are warranted for a better understanding of the association between SLE and HCM.

## Introduction

Systemic lupus erythematosus (SLE) is an autoimmune disease that can affect multiple organ systems [1]. The heart is one of the most frequently involved organs, and SLE may manifest as myocarditis, pericarditis, cardiomyopathy, arrhythmias, and coronary artery disease [2].

Hypertrophic cardiomyopathy (HCM) is a type of cardiomyopathy caused by a genetic disorder of cardiomyocytes sarcomere protein, characterized by asymmetric cardiac hypertrophy, which may result in left ventricular outflow obstruction (LVOTO) [3] that requires medical treatment with like Beta blocker, calcium channel blocker, disopyramide and mavacamten (a cardiac myosin inhibitor), or septal reduction like septal myectomy [4].

The diagnosis of HCM in adults can be established by imaging including 2D echocardiography or cardiovascular magnetic resonance (CMR) showing a maximal end-diastolic wall thickness of >15 mm anywhere in the left ventricle, in the absence of another cause of hypertrophy. More limited hypertrophy (13-) can be diagnostic of a family history of HCM or in conjunction with a positive genetic test identifying a pathogenic variant often in a sarcomere gene [4].

The prevalence of asymptomatic HCM in young adults has been reported in the range of 1:500 while symptomatic HCM has been estimated at <1:3000 adults in the United States. There is an equal distribution of HCM by sex, although women are diagnosed less commonly than men [4].

SLE cardiomyopathy is uncommon and limited to case reports. However, serious clinical manifestations occur in up to 9% of SLE patients [5]. Whether there is cardiomyopathy directly caused by lupus is not clear [6]. We present a rare case of HCM that required septal myectomy in an SLE patient.

## Case presentation

A 25-year-old Hispanic woman with a past medical history of systemic lupus erythematosus (SLE) diagnosed at the age of 20 years, end-stage renal disease (ESRD) due to lupus nephritis, epilepsy, and hypertrophic obstructive cardiomyopathy (HOCM) required left ventricular septal myectomy.

**Figure 1:**
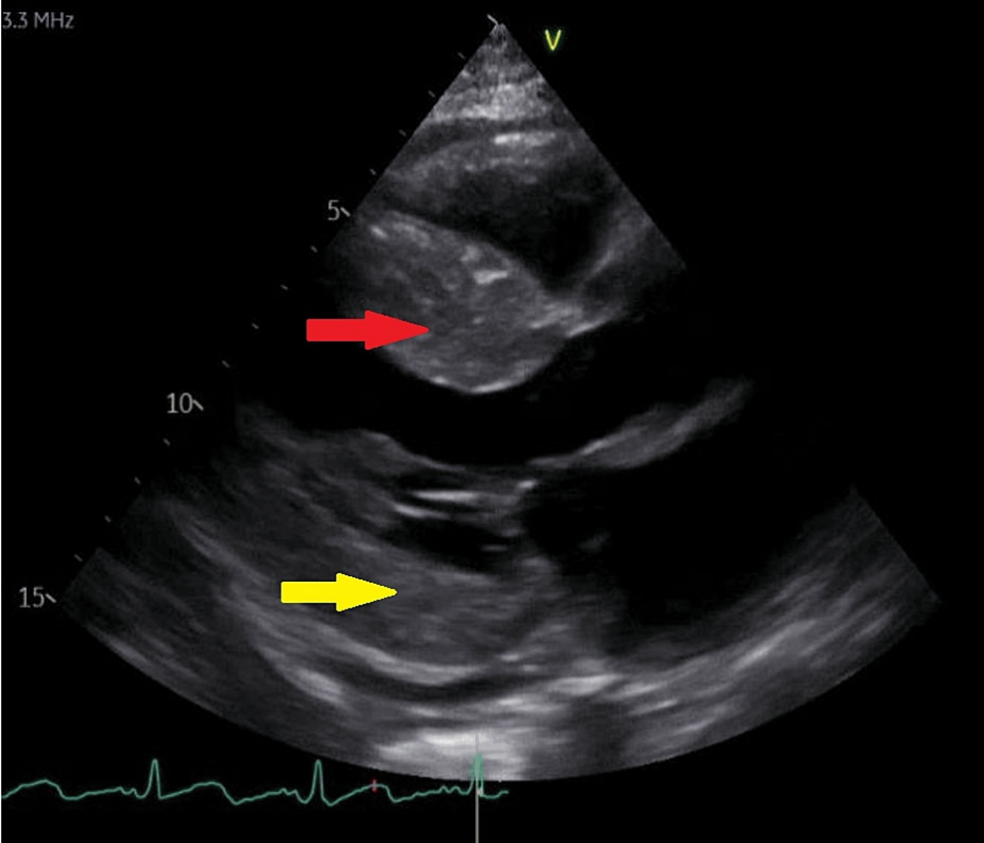
Echocardiogram showing HCM Red arrow: thickened interventricular septum, Yellow arrow: thickened posterior wall HCM: hypertrophic cardiomyopathy

She was diagnosed as HOCM by echocardiogram, which showed normal left ventricular systolic function (LVEF 73%) and dynamic flow acceleration in the left ventricular outflow tract with a gradient of more than 50 mmHg (Figure 1), then confirmed on cardiac MRI with asymmetric septal hypertrophy (maximal wall thickness 2.2 cm in the basal anteroseptum). One month ago before the current presentation, she underwent surgical LV septal myectomy to achieve septal reduction due to symptomatic LVOTO that caused multiple episodes of syncope and cardiac arrest one year ago in the setting of pulmonary edema, with good resolution of LVOTO (mean LVOT gradient post myectomy is 5 mmHg).

The initial diagnosis of SLE was in the setting of nephrotic syndrome and acute kidney injury, with positive serological tests of lupus, she had a renal biopsy consistent with class IV/V lupus nephritis. The patient was treated with IV methylprednisolone transitioned to prednisone and started on hydroxychloroquine and mycophenolate. She is currently not on any immunomodulatory therapy for SLE.

**Figure 2:**
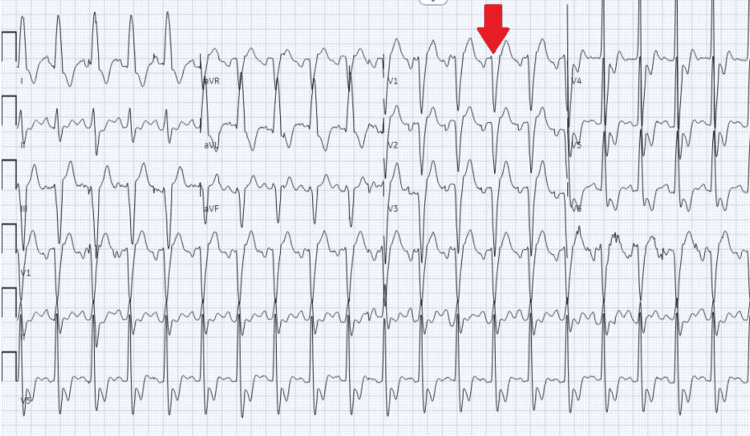
Initial ECG showing sinus tachycardia 120 beats per minute and old left bundle block morphology (red arrow)

In the current presentation, one month post-septal myectomy, she presented to the emergency department (ED) with dyspnea. She became dyspneic one day after consuming a large amount of water at home (thought to be due to psychogenic polydipsia). Her last episode of dialysis was on the day before her water binge. When she arrived at the ED, she was severely hypertensive (220/134) and tachycardic (heart rate 130 beats per minute). On examination, she was pale, diaphoretic, clammy, tachypneic, and had bilateral rales throughout the lung fields. CXR demonstrated pulmonary congestion, and EKG showed sinus tachycardia with a left bundle branch block (Figure 2).

She was given two units of non-crossmatched blood and placed on noninvasive positive pressure ventilation (NIV). Initial labs (Table 1) demonstrated elevated pro-b-type natriuretic peptide (proBNP), troponin, and lactic acidosis, along with mild anemia and leukocytosis. Electrolyte abnormalities were corrected, and she was started on piperacillin-tazobactam and vancomycin. She was administered nicardipine, with a good BP response.

**Table 1:** Selected labs on presentation BUN: blood urea nitrogen; ProBNP: pro-b-type natriuretic peptide

	Day 0	Day 3	Pre-discharge
Hemoglobin (g/dL)	10.3	8.3	8.3
WBC (/nL)	16.92	5.37	5.97
Platelets (/nL)	320	152	161
Na+ (mEq/L)	135	138	137
K+ (mEq/L)	5.3	4.0	4.6
Cl- (mEq/L)	92	96	95
CO2 (mEq/L)	22	27	26
BUN (mg/dL)	76	20	59
Creatinine (mg/dL)	7.15	3.53	5.37
ProBNP (pg/mL)	>70000		
D-dimer (ng/mL)	3114		

**Figure 3:**
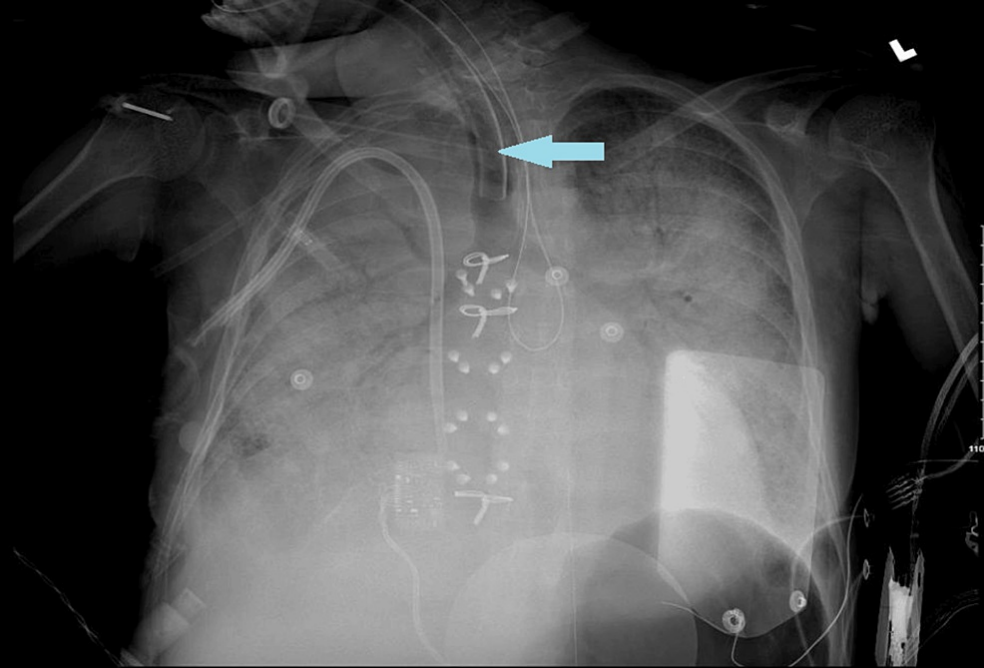
CXR on presentation (pulmonary edema); the blue arrow shows the endotracheal tube

She was, however, persistently lethargic and required intubation. The initial chest X-ray can be seen in Figure 3. She was admitted to the medical intensive care unit (MICU), and urgent dialysis was done. Intermittent hemodialysis was continued, and blood pressure (BP) control was attempted with the use of intravenous labetalol without much success. A diltiazem infusion was started with an improved response. Her MICU stay was also complicated by type 2 myocardial infarction (Table 2).

**Table 2:** Troponin trend during acute illness Trop-Hs: high-sensitivity troponin

	Day 0	Day 1	Day 2
Trop-Hs (ng/L)	63	155	142

A repeat echocardiogram in the MICU showed an ejection fraction of 65% without wall motion abnormalities, and systolic anterior motion was seen without LVOTO. She was extubated after one day and transferred to the cardiac intensive care unit (CICU) for optimization of BP control. Diltiazem was weaned, and she was restarted on a beta blocker and verapamil (her home medication). Losartan also was added for better BP control. The rheumatology team was consulted and found no evidence of an SLE activity and did not require steroid or immunosuppressive medications, she was discharged home after five days of hospitalization.

## Discussion

HCM is mainly caused by genetic mutations of the cardiac beta myosin heavy chain gene on chromosome 14 [7], but it can be associated with some diseases like Friedreich's ataxia and other neuromyopathic disorders [8] or secondary to long-term medication use such as anabolic steroids, tacrolimus, and hydroxychloroquine [9,10]. SLE, characterized by autoimmune dysregulation, can profoundly affect cardiac structures, including the pericardium, conduction system, myocardium, valves, and coronary arteries [11]. Pericarditis stands as the foremost manifestation of cardiac involvement in SLE [12]. Myocardial involvement is less common, occurring in about 9% of patients with SLE [6].

We are reporting a case of a 25-year-old female with SLE diagnosed at the age of 20 years with lupus nephritis and ESRD. In addition to steroid therapy, she was also maintained on hydroxychloroquine initially after diagnosis, but it was discontinued after three years of use. She was found to have HCM initially on echocardiogram (Figure 3), and this was confirmed on cardiac MRI with asymmetric septal hypertrophy (maximal wall thickness 2.2 cm as measured in basal anteroseptum). TTE showed upper limit normal left ventricular systolic function (LVEF 73%) and dynamic flow acceleration in the left ventricular outflow tract with a gradient of more than 50 mmHg despite maximal medical therapy. Later she underwent a septal myectomy. She had no family history of cardiac disease, and no genetic testing was pursued.

To date, only 10 cases of concomitant SLE with HCM have been reported. Among these cases, nine cases (90%) were female, with a mean age of 36.0 ± 10 years, and had a history of systemic disease for many years [13-16].

The existing literature endeavors to elucidate the potential relationship between SLE and HCM. In a case study documented by Ma et al., genetic analysis was done to rule out primary HCM, aiming to discern an alternative etiological pathway connecting SLE and HCM. However, ambiguity persists regarding whether this was attributable to SLE itself or the medication used in the management of SLE [16]. Fiorito et al. proposed a plausible link between the manifestation of HCM and the HLA-DR3 alloantigen. Notably, this antigen, alongside HLA-DR2, exhibits a well-established association with SLE, particularly among individuals of European descent [17]. Nevertheless, further studies are warranted for a deeper comprehension of the association between SLE and HCM.

## Conclusions

We are reporting a rare case of SLE complicated with ESRD and associated with HOCM that required lLV septal myectomy. Although a co-occurrence of SLE and HCM was reported in approximately 10 cases, the underlying mechanisms linking SLE and HCM remain unclear. Notably, there is a significant gender disparity with 90% of these cases occurring in females. Further research is warranted for a better understanding of the relationship between SLE and HCM.

## References

[1] Oku K, Atsumi T (2018). Systemic lupus erythematosus: nothing stale her infinite variety. Mod Rheumatol.

[2] Ming Wang TK, Chan N, Khayata M (2022). Cardiovascular manifestations, imaging, and outcomes in systemic lupus erythematosus: an eight-year single center experience in the United States. Angiology.

[3] Marian AJ, Braunwald E (2017). Hypertrophic cardiomyopathy: genetics, pathogenesis, clinical manifestations, diagnosis, and therapy. Circ Res.

[4] Ommen SR, Ho CY, Asif IM (2024). 2024 AHA/ACC/AMSSM/HRS/PACES/SCMR guideline for the management of hypertrophic cardiomyopathy: a report of the American Heart Association/American College of Cardiology Joint Committee on Clinical Practice Guidelines. Circulation.

[5] Gottenberg JE, Roux S, Assayag P, Clerc D, Mariette X (2004). Specific cardiomyopathy in lupus patients: report of three cases. Joint Bone Spine.

[6] Ishimori ML, Agarwal M, Beigel R, Ng RK, Firooz N, Weisman MH, Siegel RJ (2014). Systemic lupus erythematosus cardiomyopathy—a case series demonstrating a reversible form of left ventricular dysfunction. Echocardiography.

[7] Jarcho JA, McKenna W, Pare JA (1989). Mapping a gene for familial hypertrophic cardiomyopathy to chromosome 14q1. N Engl J Med.

[8] Child JS, Perloff JK, Bach PM (1986). Cardiac involvement in Friedreich's ataxia: a clinical study of 75 patients. J Am Coll Cardiol.

[9] Joyce E, Fabre A, Mahon N (2013). Hydroxychloroquine cardiotoxicity presenting as a rapidly evolving biventricular cardiomyopathy: key diagnostic features and literature review. Eur Heart J Acute Cardiovasc Care.

[10] Noda K, Ukichi T, Furuya K, Yoshida K, Kingetsu I, Tanaka T, Kurosaka D (2017). Tacrolimus-induced hypertrophic cardiomyopathy in a patient with dermatomyositis. Rheumatology (Oxford).

[11] Moder KG, Miller TD, Tazelaar HD (1999). Cardiac involvement in systemic lupus erythematosus. Mayo Clin Proc.

[12] Mohamed AA, Hammam N, El Zohri MH, Gheita TA (2019). Cardiac manifestations in systemic lupus erythematosus: clinical correlates of subclinical echocardiographic features. Biomed Res Int.

[13] Kotani T, Takeuchi T, Sakamoto M (2005). A case of systemic lupus erythematosus associated with hypertrophic cardiomyopathy [Article in Japanese]. Nihon Rinsho Meneki Gakkai Kaishi.

[14] Ara J, Vivancos J, Soler-Carrillo J, Paré JC, Cervera R, Font J (1998). Hypertrophic cardiomyopathy and systemic lupus erythematosus. Clin Rheumatol.

[15] Anastasiadis GP, Moyssakis I, Boki K, Kyriakidis M (2001). Hypertrophic cardiomyopathy in systemic lupus erythematosus. Mayo Clin Proc.

[16] Ma H, Cao X, Zhang J (2021). Systemic lupus erythematosus complicated with hypertrophic cardiomyopathy: a case report and literature review. Case Rep Cardiol.

[17] Fiorito S, Autore C, Fragola PV (1986). HLA-DR3 antigen linkage in patients with hypertrophic obstructive cardiomyopathy. Am Heart J.

